# Analyzing the Dietary Diary of Bumble Bee

**DOI:** 10.3389/fpls.2020.00287

**Published:** 2020-03-25

**Authors:** Robert M. Leidenfrost, Svenja Bänsch, Lisa Prudnikow, Bertram Brenig, Catrin Westphal, Röbbe Wünschiers

**Affiliations:** ^1^Department of Biotechnology and Chemistry, Mittweida University of Applied Sciences, Mittweida, Germany; ^2^Functional Agrobiodiversity, Department of Crop Sciences, University of Göttingen, Göttingen, Germany; ^3^Institute of Veterinary Medicine, University of Göttingen, Göttingen, Germany

**Keywords:** biodiversity, ecology, pollen, bumble bee, ITS2, next-generation sequencing, third-generation sequencing, Nanopore

## Abstract

Bumble bees are important crop pollinators and provide important pollination services to their respective ecosystems. Their pollen diet and thus food preferences can be characterized through nucleic acid sequence analysis. We present ITS2 amplicon sequence data from pollen collected by bumble bees. The pollen was collected from six different bumble bee colonies that were placed in independent agricultural landscapes. We compared next-generation (Illumina), i.e., short-read, and third-generation (Nanopore), i.e., MinION, sequencing techniques. MinION data were preprocessed using traditional and Nanopore specific tools for comparative analysis and were evaluated in comparison to short-read sequence data with conventional processing. Based on the results, the dietary diary of bumble bee in the studied landscapes can be identified. It is known that short reads generated by next-generation sequencers have the advantage of higher quality scores while Nanopore yields longer read lengths. We show that assignments to taxonomic units yield comparable results when querying against an ITS2-specific sequence database. Thus, lower sequence quality is compensated by longer read lengths. However, the Nanopore technology is improving in terms of data quality, much cheaper, and suitable for portable applications. With respect to the studied agricultural landscapes we found that bumble bees require higher plant diversity than only crops to fulfill their foraging requirements.

## Introduction

Crop pollinators such as wild and domestic bees are important ecosystem service providers and in high demand (Aizen et al., 2008). The pollination services rendered by these pollinators are affected by changes to floral resources in semi-natural habitats and simplification of agricultural landscapes (Steffan-Dewenter and Westphal, 2008). Intensification of agricultural land use at local and landscape scales is considered as one major driver of pollinator declines due to shortages in the supply with pollen and nectar resources (Potts et al., 2010; IPBES, 2019). To sustain future crop pollination services in changing agricultural landscapes, it is important to characterize the foraging ecology of wild and domestic bees. Bumble bees are important crop pollinators because of their general floral diets and their large foraging ranges (Westphal et al., 2006; Kleijn et al., 2015).

We aim to identify the pollen diet of a common bumble bee species (*Bombus terrestris* L.) in agricultural landscapes. In this respect, the identification of pollen resources can reveal part of their food plant preferences and dietary requirements and thus can guide future conservation measures and EU agri-environmental schemes. Identification and quantification is generally possible by labor-intensive pollen microscopy (Marzinzig et al., 2018) or nucleic acid sequence analysis (Danner et al., 2016). Approaches to the latter include DNA barcoding (Taberlet et al., 2012; Sickel et al., 2015; Bell et al., 2016) and genome skimming (Dodsworth, 2015). Most recently, a semi-quantitative approach involving Nanopore sequencing has been reported (Peel et al., 2019). The internal transcribed spacer (ITS) sequence is a popular genetic species barcode in plants (Chen et al., 2010; Yao et al., 2010; Bell et al., 2016).

In this study, we are sequencing ITS2-derived amplicons from plant pollen collected by bumble bees in order to identify pollen source species. From this data we derive bumble bees’ pollen foraging under given environmental settings using a geographically customized BLAST database derived from the ITS2 database (Merget et al., 2012). Since ITS2-amplicons generated with common primer pairs typically exceed the length of polymerase-derived NGS-reads, we are evaluating full-length MinION-based ITS2-amplicon sequencing in contrast to NextSeq-based sequencing. From a technical perspective this work aims at developing field protocols for a rapid MinION-based assessment of pollen plant diversity in the field and utilization by pollinators, including estimation of crop pollination services delivered (Pomerantz et al., 2018; Krehenwinkel et al., 2019).

## Materials and Methods

**FIGURE 1:**
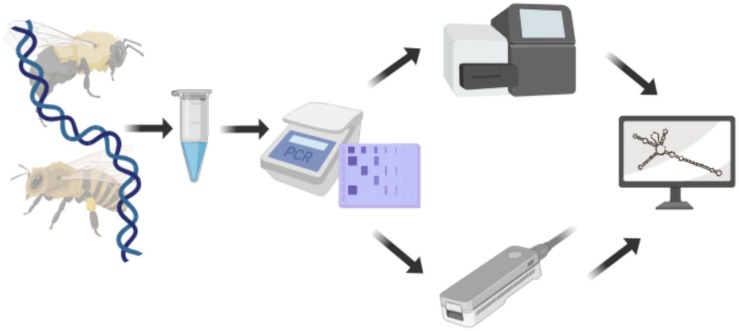
Experimental setup used to compare Illumina and Nanopore sequencing technologies. DNA was extracted from pollen and the ITS2 region amplified. Amplicons were sequenced with either, Illumina NextSeq or Nanopore MinION sequencer before being subjected to analysis. Created with biorender.com.

Pollen-DNA extracts were PCR-amplified with ITS2-specific primers. Amplicons were then sequenced on NextSeq and MinION platforms, respectively (Figure 1).

### Pollen Collection

Pollen was collected from bumble bees in front of their hives between May and June 2017. The bumble bee colonies were purchased from a German bumble bee breeder (STB Control, Aarbergen, Germany). The hives were located close to commercial strawberry fields (Supplementary Material S1). Pollen loads were collected by capturing, if possible, five individual bees in front of their colonies with an insect net. Pollen was removed from the hind tibia with tweezers. Afterward, bumble bees were released. We pooled the pollen loads of each observation date by colony and homogenized them in 70% (v/v) ethanol [one part pollen and four parts 70% (v/v) ethanol]. We prepared 1 mL aliquots for microscopic (not shown) and molecular pollen analysis by centrifugation for 10 min at 15,400 × *g*. We then removed the supernatant and dried them for 72 h in a clean bench.

### Nucleic Acid Extraction

The DNA of approximately 0.015 g pollen aliquots was isolated using the DNeasy Plant Mini Extraction Kit from Qiagen according to the manufacturer’s instructions. Cell lysis and homogenization of the samples were modified as follows: 150 g ceramic beads (1.4 mm), one tungsten carbide bead (3 mm), and 200 μL buffer AP1 were added to each dried sample. Samples were homogenized twice with a FastPrep Instrument (FastPrep^®^ FP120, ThermoSavant) for 45 s at 6.5 m/s with a cooling step with ice in-between. Another 200 μL buffer AP1 were added. Finally, the standard protocol was followed until the DNA was eluted with 50 μL of elution buffer. DNA concentration and quality were measured using a Nanodrop (Thermo Fisher Scientific, Massachusetts, United States), and, prior to MinION Nanopore sequencing, with Qubit 3.0, dsDNA HS Assay Kit (Invitrogen, Eugene, United States).

### ITS2 Amplicon Generation

For each sample, we performed three separate 10 μL PCR reactions to reduce PCR bias (Sickel et al., 2015) using the primers ITS2F [ATGCGATACTTGGTGTGAAT; Tm 61°C (Chen et al., 2010)] and ITS4R [TCCTCCGCTTATTGATATGC; Tm 60°C (White et al., 1990)]. Each reaction contained 0.3 μL FastStartTaq Polymerase (5 U/μL, Roche, Mannheim, Germany), 0.5 μL dNTPs (0.5 mM), 0.75 μL of each forward and reverse primer (10 pmol/μL), 2.5 μL 10× PCR buffer with MgCl_2_ at a concentration of 20 mM (Roche, Mannheim, Germany), 19.2 μL PCR grade water, and 1 μL DNA template. The PCR conditions were optimized to the following conditions: initial denaturation at 95°C for 10 min, 37 cycles of denaturation at 95°C for 40 s, annealing at 49°C for 40 s, and elongation at 72°C for 40 s. Final extension was performed at 72°C for 5 min.

All reactions were checked for successful amplifications and contaminations by gel electrophoresis (1.5% agarose gels stained with ethidium bromide, 120 V for 30 min). Triplicate PCR products were pooled per sample and purified using the QIAquick PCR Purification Kit (QIAGEN, Hilden, Germany).

### NextSeq500 Illumina Sequencing

Paired-end sequencing (2 × 150 bp) was performed on a NextSeq500 platform (Illumina, San Diego, CA, United States) using a Mid-output flowcell (150 cycles). Of each amplicon 500 ng was used for library preparation according to the manufacturer’s protocol (NEBNext Ultra II DNA Library Prep Kit for Illumina, New England Biolabs, Munich, Germany).

### MinION Nanopore Sequencing

Nanopore sequencing of each amplicon was performed using the MinION [Oxford Nanopore Technologies (ONT), Oxford, United Kingdom] and 1D native barcoding according to protocols (EXP-NBD103 and SQK-LSK108, ONT; NEBNext End repair/dA-tailing Module, NEB Blunt/TA Ligase Master Mix, NEBNext Quick Ligation Module, New England Biolabs, Munich, Germany; AMPure XP beads, Agencourt) on a R9.4.1 flow cell (FAH89141, ONT, run QC = 1253 pores). Shearing and DNA repair steps were omitted. Incubation times during end-prep step were prolonged to 20 min. At designated checkpoints during library preparation, DNA was quantified using Qubit 3.0 fluorometer (dsDNA HS Assay Kit, Invitrogen, Eugene, United States). Data acquisition was performed by MinKNOW (v_1.15.6, ONT) and subsequent base-calling by Albacore (v_2.3.4, ONT).

### Data Analysis

Basecalled MinION data were demultiplexed using Porechop (v_0.2.4, no further parameters set^1^) and assessed by NanoPack [v_1.13.0; Nanoplot 1.27.0 (Coster et al., 2018)]. A cursory look into the data was performed using Kraken2 [v_2.0.7-beta; NCBI non-redundant nucleotide database built in 2018-09 (Wood et al., 2019)] and subsequent visualization with Krona (Ondov et al., 2011). Reads were further processed by removing primers, using either USEARCH [v_11.0.667_i86linux32 (Edgar, 2010)] or Porechop containing ITS2F and ITS4R primer sequences.

In order to increase the accuracy of assignment of amplicon reads to plant-specific ITS2 sequences, we extracted all ITS2 sequences from a global eukaryota database (Ankenbrand et al., 2015) for plants that have previously been detected in Lower Saxony, Germany (Garve, 2004, 2007). The resulting subset was made non-redundant by clustering identical entries with VSEARCH (Version 2.9.1; Rognes et al., 2016) and subsequently used to create a magicBLAST database (version 1.4; Boratyn et al., 2018). After querying the Illumina amplicon reads against this database, all paired reads that both aligned to the same plant ITS2 sequence database entry with at least 50 bp each and a similarity greater than 98% were kept.

For each matching read, we calculated an alignment quality score by multiplying the alignment length with the alignment identity, thus accounting for overall alignment quality. The scores for the forward and reverse read were summed to get a final score for each read-pair. Read-pairs that matched several entries were ordered by this score. Only the top scoring match (plant species) per read was counted. As some plant species have very similar ITS2 sequences and we, therefore, cannot unambiguously distinguish them on a species level, we decided to use all sequence data down to the genus level only. If there were more than one scoring match with an identical score, we decided on a match with higher reliability based on personal observations in the field, flowering time and a distribution atlas of plants in Lower Saxony (Garve, 2007). The final alignment quality score assigned to each read, respectively, was used for taxonomic assignment. Ultimately, pollen richness was calculated as the amount of plant genera in the respective pollen sample.

## Results and Discussion

**FIGURE 2:**
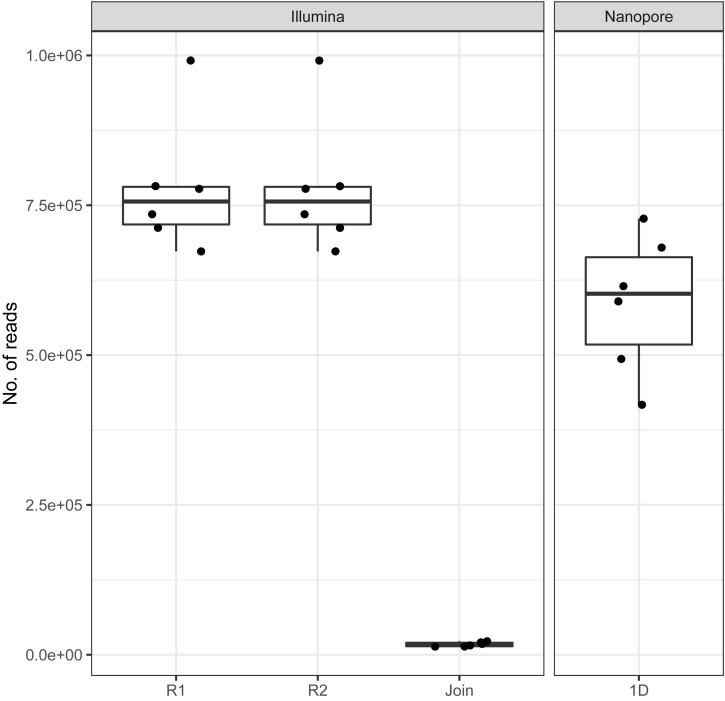
Amount of reads generated with Illumina and Nanopore sequencers across all six samples. Note the low amount of joinable sequences for the Illumina data as a result of amplicon length > 300 bp.

**FIGURE 3:**
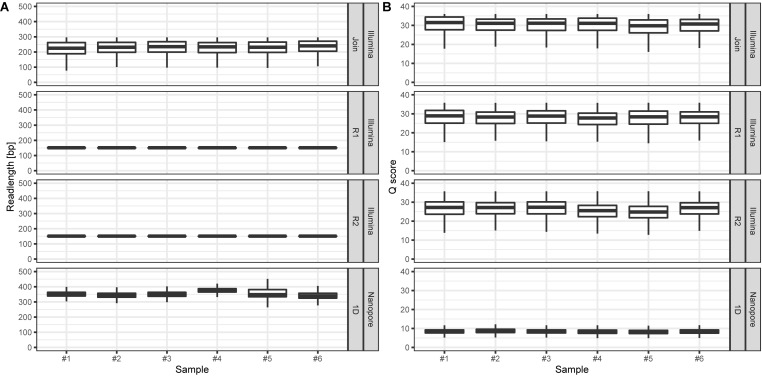
**(A)** Read lengths [bp] for Illumina (min: 25, max: 296, figure split by R1, R2, and joined reads, respectively) and Nanopore (min: 5, max: 11,519) data per sample. **(B)** Corresponding mean read Q scores for Illumina (median: 27.5) and Nanopore (median: 8.5) data per sample. Outliers are not displayed.

On average, we retrieved 778,566 reads from the NextSeq and 588,252 reads from the MinION platform, respectively (Figure 2). While the read length was fixed to 150 nt by the Illumina chemistry, Nanopore reads varied from 340 to 380 nt with an average of 354 nt, after trimming with Porechop (Figure 3A). Generally, trimming reduced the average length of a MinION read by 25%, while at the same time increasing the average read quality score by 3.5%. A full length native barcode adapter, as identified by Porechop, is of ∼65 nt length, with the actual barcode consisting of 24 nt. Our trimming approach (using default parameters) resulted in the least removal of problematic artifacts and was made intentionally to establish a baseline. It may of course be made more stringent through more careful filtering before proceeding with downstream analysis, solving potential inaccuracies and circumventing technical artifacts (White et al., 2017; Xu et al., 2018).

With Nanopore technology being capable to generate sequence reads of several thousand nucleotides, the resulting average of 354 nt resembles full amplicon length (Figure 3A). Hence, it can be concluded that Illumina, even with paired-end sequencing, would not cover the whole amplicon, whereof 2 × 40 nt account for the forward and reverse primer, respectively. Even the sequencing kit for 300 cycles, at almost twice the cost, would be insufficient to provide full-length amplicon reads. Plant ITS2-sequences may exceed 600 nt (Yao et al., 2010). Therefore, only 3%, i.e., in average 17,406 of the paired-end reads, could be joined with standard bioinformatic tools [FastQ-join (Aronesty, 2013)] to full amplicon reads (Figure 2). Hence, we developed a magicBLAST pipeline as described in the methods to assign unjoined reads to target plants.

We observed only a fraction (5.9%) of Illumina reads that were shorter than the expected 150 nt. In contrast, Nanopore reads had a wider length variability (Figure 3A, min: 5bp; median: 350 bp; max. 11,519 bp), probably reflecting (a) varying ITS2-sequence sizes (Yao et al., 2010), (b) incomplete and/or unspecific amplicons, and (c) library preparation artifact. The latter is most likely based on the library preparation ligation protocol, since randomly picked long reads turned out to be concatenated amplicons.

**FIGURE 4:**
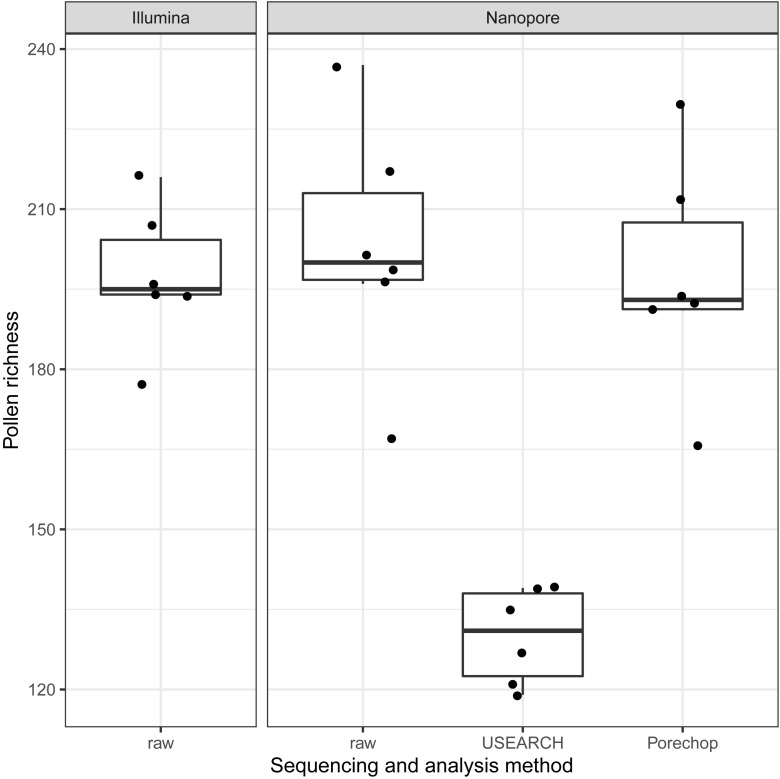
Boxplot-comparison of assigned genera for Illumina and Nanopore reads, respectively. Pollen richness corresponds to the number of genera detected when BLAST querying against the Lower Saxony specific ITS2-sequence database. USEARCH’s amplicon extraction led to less assignments than Porechop’s read-end trimming.

The amplicon read mean quality scores (Phred score) were averaging around 30 for NextSeq data, which is approximately 15 to 20 units higher compared to the MinION data (Figure 3B). While the quality of reads generated by Nanopore sequencer technology can be expected to improve due to technical optimizations, at the current technical level, short reads generated by next-generation sequencer technology such as provided by Illumina are of better quality (Rang et al., 2018; van Dijk et al., 2018). The lower average read quality scores of the sequence reads generated by Nanopore MinION reflect its error prone nature. This is especially the case with the flanking regions containing the 20 nt primer sequences. Those contained up to 30% single nucleotide mismatches. Yet, Nanopore reads can still be BLAST-assigned to the ITS2-sequence database to a similar extent as Illumina reads: Average pollen richness, i. e. assigned genera, for Illumina reads is 197 (min.: 177; max.: 216). For Nanopore reads the average pollen richness is 203 (min.: 167; max.: 237) (Figure 4). Primer clipping with Porechop does hardly change the mean pollen richness, albeit a wider span is observable (mean: 198; min.: 166; max.: 230). In contrast, amplicon extraction with USEARCH reduces the number of assignments (mean: 130; min.: 119; and max.: 139).

**FIGURE 5:**
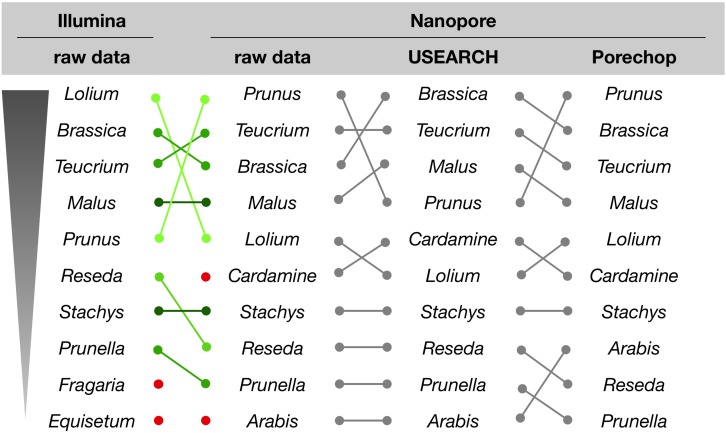
Genus assignment for sample #6 by querying raw or processed sequence reads against the Lower Saxony specific ITS2-sequence database. Only the ten most abundant genera are shown in descending order, as indicated. Lines represent different ordering; red dots indicate genera that appear further down in the list.

For the initial comparison of sequencing technologies as presented in this study, we focused on a qualitative rather than quantitative analysis of the assignment results. With respect to the genus assignments performed on NextSeq and MinION data, the sample with the most divergent ranking (sample #6), is differing only in the order, but not the presence of the ten most abundant genera (Figure 5). This result is supported by a microscopic analysis of pollen grains (not shown).

**FIGURE 6:**
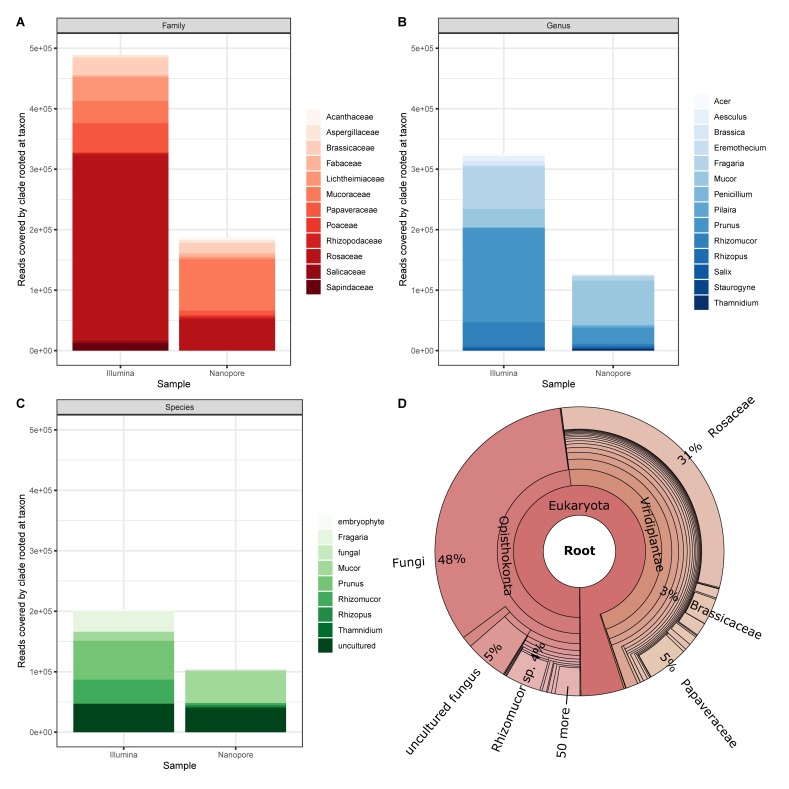
Cursory display of the ten most abundant assignments from Kraken2 reports for sample #6 data generated by Illumina and Nanopore platforms, respectively on **(A)** Family, **(B)** Genus, and **(C)** Species level. **(D)** Krona plot for the Illumina data (17 levels displayed).

Finally, we applied an assignment approach without the application of BLAST and our Lower Saxony specific ITS2-sequence database. Instead we used Kraken2 that queries for exact k-mer sequence matches in a k-mer database that is based on NCBIs non-redundant nucleotide DB. This approach achieves high accuracy with fast classification speed (Wood and Salzberg, 2014; Wood et al., 2019). Again, to establish a baseline, we focus on the sample that generated most divergent results between Illumina and Nanopore data (sample #6, Figure 6). Prominently, taxonomic units other than plants are detected as a result of the DB employed by Kraken2. This “bycatch” constitutes representatives from the kingdoms fungi and – in lower abundance – metazoa and bacteria. While caution must be taken when interpreting this finding in detail for the Nanopore data due to their error-prone nature, the detection of especially fungal species was also clearly visible in the Illumina data visualized with Krona (Figure 6D). Indeed, the presence of molds in pollen is not uncommon (Kacániová et al., 2009; Belhadj et al., 2015; Nardoni et al., 2016). Moreover, despite Nanopore yielding less than half of the total reads (Illumina ∼990k reads, Nanopore ∼417k reads), Kraken2 assigned those reads to roughly twice the assigned genera (Illumina ∼3,648 genera, Nanopore ∼1,731 genera). This is, again, most likely due to (a) the error prone nature of Nanopore reads (Rang et al., 2018), and (b) the much larger database size (NCBI non-redundant nucleotide k-mer database versus Lower Saxony plant specific ITS2-sequences). Ultimately, the choice for either approach, ITS2 versus Kraken2, depends on the research purpose.

In terms of bumble bee foraging in different agricultural landscapes, our results show that colonies are not only heading to the close strawberry field (*Fragaria*). Instead, also plants of the genus *Brassica*, which is most likely oilseed rape because it is flowering intensively in May in the investigated regions, and flowers of a great variety of other plant genera were visited (Figure 6B). Beside the annual crops (e.g., oilseed rape and strawberry) in the agricultural landscape matrix, bumble bees also visited woody structures such as *Prunus* and *Acer*. Cherry trees belong to the genus *Prunus* and are commonly found in home gardens but also along roadsides. The same is true for *Acer*, *Aesculus* (chestnut), and *Salix* (willow), which are common trees in agricultural and urban areas. Our findings indicate that bumble bees visit much more plants genera than only crops in the agricultural landscape to fulfill their foraging requirements. High pollen diversity is likely to promote colony performance (Hass et al., 2019). Furthermore, bumble bees potentially pollinate not only crops but also many wild plant species. Interestingly, we also detected a large number of sequences derived from fungi (Figures 6B,D), which may inhabit flowers (Keller et al., 2015).

We like to mention that the bumble bee samples used for this comparison of sequencing methods are part of a larger study that investigates pollen resource usage of bumble bees in more detail, including a comparison to honey bee foraging and with respect to landscape parameters (Bänsch et al., submitted). The primary focus of the study presented here is the comparison of the applicability of third-generation nanopore sequencing in contrast to established next-(second-)generation sequencing methods. Obviously, both technologies have their strengths and weaknesses. While MinION and NextSeq perform comparably well when querying against an ITS2-specific sequence database, shorter genetic markers still benefit from the higher accuracy of next-generation sequencing.

## Conclusion

The goal of our study is to compare polymerase (Illumina NextSeq) and nanopore (Oxford Nanopore Technology MinION) generated sequence reads for the assignment of pollen DNA to plant genera. Illumina reads have the advantage of higher quality scores. In contrast, the Nanopore sequencing technology yields longer read lengths. Starting with ITS2-amplicons, we employed two different assignment approaches: (a) BLASTing against a Lower Saxony specific ITS2-sequence database (created within this study) and (b) querying against a k-mer genome sequence database with Kraken2. For (a) the results are comparable: the lower sequence quality is compensated by the read length. For (b) there are two observations striking: (i) the identification of “bycatch” depicted as result of the more extensive database and (ii) the higher amount of assigned taxonomic units on genus level despite the overall smaller read dataset, most likely reflecting the error prone nature of nanopore reads.

In conclusion, we demonstrate the applicability of MinION nanopore sequencing analyzing the dietary diary of bumble bee. Sequence read processing with open software tools and standard parameters yield results close to established next-generation sequencing.

## Data Availability Statement

The datasets generated for this study can be found at bioproject accession PRJNA593728 (https://www.ncbi.nlm.nih.gov/bioproject/PRJNA593728). Under Github repository https://github.com/awkologist/BumbleBeeDietaryDiary the ITS2 database used in this study is freely accessible.

## Author Contributions

SB, CW, and RW conceived the study. SB conducted the field work. SB, BB, RL, and LP performed the DNA extraction and sequencing. RL, LP, and RW analyzed the data. RL and RW wrote the manuscript. All authors reviewed and revised the manuscript.

## Conflict of Interest

The authors declare that the research was conducted in the absence of any commercial or financial relationships that could be construed as a potential conflict of interest.

## Footnotes

1 https://github.com/rrwick/Porechop
