# Supplementary material for: Analyzing the Dietary Diary of Bumble Bee
Source: Front Plant Sci. 2020 Mar 25;11:287. doi: 10.3389/fpls.2020.00287 (PMC7109327; doi:10.3389/fpls.2020.00287)
Supplement: Supplementary file 1 [file Table_1.pdf]

Supplementary Material

1 Experimental setup of bumble bees in the field

Bumble bee colonies were placed adjacent to five commercial strawberry fields (*Fragaria* x *Ananassa*) in southern lower Saxony and Northern Hesse. Surrounding landscape was mapped using a geographic information system (ESRI ArcGIS, Version 10.3.1) within a radius of 2000 m around the strawberry fields. The range of 2000 m is a common foraging range of *B. terrestris* (Westphal et al., 2006). The surroundings are characterized by an agricultural landscape matrix (61.86 % ± 9.03), semi-natural habitats (6.28 % ± 0.69), urban area (21.62 % ± 6.96) and forests (6.78 ± 1.96). The agricultural landscape matrix includes flowering crops such as oilseed rape and strawberry but also diverse resources along tree lines or roadsides. Semi-natural habitats include for example hedges or meadow orchards. While urban area consists of a large amount of concealed area, it can provide many resources in gardens and parks as well.

References

Westphal, C., Steffan-Dewenter, I., and Tscharntke, T. (2006). Bumblebees experience landscapes at different spatial scales: possible implications for coexistence. *Oecologia* 149, 289–300.
